# 

**Published:** 1882-08-20

**Authors:** 


### TABLE OF CONTENTS.

ORIGINAL AND SELECTED ARTICLES.

Clinical Report of a Compound Comminuted Fracture, etc., by Drs. Hayes Bros., Marianna, Ark., 281. Iodoform as a Dressing to Fresh Wounds, by Lindsay Johnson, M. D., of Ga., 285. Gonorrhœa, by T. H. Logan, M. D., of Ga., 287. The Differential Diagnosis of the Cause of Sudden Unconsciousness, by R O Beard, M.D., 288. Cholera Infantum, by W. F. Hamer, M. D., 292. Hydrocyanate of Iron in the Treatment of Neuralgia, by Charles K. Gardner, M. D., Laurinburg, N. C., 295. Remedies for Sleeplessness, by W. E. Green, M. R. C. S., Eng., 296.

ABSTRACTS AND GLEANINGS.

Hot Water in Therapeutics, 297. The Bromides, 298. Koch at the German Congress for Internal Medicines, 300. New Method of Inducing Sleep, 301. Vesico Vaginal Fistula, 302. How to Count a Rapid Pulse, 303. Sunstroke; Where is a Man's Stomach? 304. Elixir Iodo, 305. Digestive Wine; Two Hundred and Fifty Cases of Malaria Treated, etc., 306. Lutidine as an Antidote for Strychnia; Criteria of Insanity, 307. Bacilli in Tubercle; Calabar Bean in Obstinate Constipation; Agaricus in the Treatment of Night Sweating, 308. Congress of German Surgeons; The Effect of Thapsia Garganica on the Skin, 309. The Influence of Certain Remedies upon the Milk Secretion; Jaborandi; Sensible, 310.

SCIENTIFIC ITEMS.

Brown Sugar Under the Microscope; Transmission of Motive Power, 311. Photo Printing in Colors; Dynamite, 312.

PRACTICAL NOTES AND FORMULÆ.

In Dyspepsia with Acid Eructations and Debility; In Pyrosis and Gastrodynia; Camphorated Chloro-Tannate of Iodine; Injection for Sciatica; Treatment of Phagedenic Ulcers, 313. Incontinence of Urine; In Heart-Burn and Acid Eructations; Iodoform in Ulcers; Purpura; Dyspnœa of Phthisis and Emphysema, 314. Iodoform in Lung Disease; Prevention of Diphtheritic Infection, 315.

EDITORIALS AND MISCELLANEOUS.

The Red Cross; Surgeon General of the U. S.; Prevalence of Cancer; Death of Dr. A. S. Heaton, 316. The Texas Pharmaceutical Association; Gynecological; Cincinnati Sanitarium; Celerina; Book Notices, 317. Receipted; Special Notices 320.

